# Emotional Availability, Neuropsychological Functioning, and Psychopathology: The Context of Parental Substance Use Disorder

**DOI:** 10.1155/2018/5359037

**Published:** 2018-05-17

**Authors:** Alessio Porreca, Zeynep Biringen, Micol Parolin, Hannah Saunders, Giulia Ballarotto, Alessandra Simonelli

**Affiliations:** ^1^Department of Developmental and Social Psychology, University of Padua, 35131 Padua, Italy; ^2^Department of Human Development & Family Studies and Colorado School of Public Health, Colorado State University, Fort Collins, CO 80523, USA; ^3^Department of Clinical and Dynamic Psychology, Sapienza University of Rome, 00185 Rome, Italy

## Abstract

Parental Substance Use Disorder (SUD) constitutes a high-risk condition for parent-child interactions and child development. Empirical evidence indicates high rates of psychopathology and neuropsychological impairments in individuals with SUD. Despite research indicating that parenting skills are related to psychological well-being and cognitive/neuropsychological functioning, prior studies have not examined the associations between these areas of parental functioning and the quality of parent-child interactions in the context of SUD.* Aim(s)*. The present study adopts an integrated perspective to investigate the way in which maternal neuropsychological functioning and psychopathology are associated with mother-child emotional availability (EA), in the context of parental Substance Use Disorder.* Methods*. Twenty-nine mothers with SUD were assessed in interaction with their children, as well as with respect to their neuropsychological functioning and psychopathology.* Results*. In this group, high rates of maternal neuropsychological impairments and psychopathology, as well as generally low levels of EA, were uncovered. Regression analyses showed that maternal neuropsychological functioning was significantly associated with mother-child EA, specifically sensitivity; the role of maternal psychopathology, however, was only marginally significant.* Conclusion*. In the context of SUD, maternal neuropsychological impairments are significantly associated with mother-child EA. Clinical implications of the findings are discussed.

## 1. Theoretical Background

Parenting encompasses a wide range of behaviors and emotions, including accurate perception of child cues and appropriate responsiveness to them; provision of protection and nurturance in times of need; understanding of the child's unique perspective in different situations and at different ages; and the nuanced expression of parental love, acceptance, and commitment [1–4]. Parenting behaviors are guided both by one's past experiences as a child and by actual experiences with the baby, which are observed during everyday interactions [5]. Parents' psychological well-being plays a crucial role in determining the quality of such behaviors [6]. Research indicates that the presence of psychopathological symptoms in the parent, such as anxiety or depression, strongly correlates with less positive parent-child interactions [7, 8].

Moreover, recent research on parenting highlights the strong associations that exist between parenting skills and parents' cognitive functioning, suggesting that adult neuropsychological functioning could be associated with observed caregiving [9, 10]. Within the more global domain of parental cognitive functioning, particular attention has been given to executive functioning (EF). EF is considered responsible for different cognitive processes, such as inhibition, attention, cognitive flexibility, planning, and emotion regulation [11]. These higher-level cognitive processes are responsible for the control and the regulation of lower-level processes (i.e., emotions and behaviors), and they help to establish connections among the inputs, internal states, and outputs that are needed to achieve specific goals [12]. As such, an individual's EF is important for interacting with a child in a sensitive manner, for regulating one's emotions during challenging situations, and for helping the child do so, as well as for making everyday sound decisions [13, 14]. Parents with higher EF tend to be more warm, sensitive, responsive, and flexible in interactions with their children than those with lower EF [14, 15]; additionally, parents with lower EF tend to be less positive and less capable in managing intense emotions than those with higher EF [10]. Finally, some studies report associations between high parental EF and parental psychological well-being [16–18].

In the context of high-risk parenting, maternal Substance Use Disorder (SUD) is widely recognized as a condition that profoundly interferes with parenting functions and child development [19–22]. More specifically, researchers have suggested that substance addiction exerts a specific impact on parenting, modulating the reward and stress circuits responsible for the neurobehavioral networks of parenting [23]. In the condition of SUD, the reward system is coopted by drugs, with the purpose of maintaining addictive behaviors. In turn, these behaviors become progressively more strongly associated with the relief of stress and negative emotions, making other social stimuli less rewarding [23]. As a consequence, infant stimuli become less salient for parents with SUD and can be instead perceived as stressful, [24], rather than being part of a mutually rewarding system of positive exchanges. Mothers with SUD have been found to interact in less sensitive, more intrusive, and, often, more intensely hostile ways during mother-child interactions, as compared to mothers without this diagnosis [25–28]. In addition, children exposed to substances in utero often present as more irritable and with difficulties with emotion regulation [25, 29], as compared to children who have not been exposed to substances in utero. Such challenging behaviors on the part of the child have the potential to evoke negative emotional reactions within a vulnerable parent and thereby disrupt the predictability and organization typically seen in more healthy relationships [30].

Individuals with SUD often report symptoms in additional areas of psychopathology, suggesting that there is significant comorbidity of substance use with other areas of dysfunction [31, 32]. SUD has been linked with the Anxiety Disorders [33], Major Depressive Disorder [34], Bipolar Disorder [35], and Posttraumatic Stress Disorder [36], as well as sleep disturbance [37] and even suicide attempts [38]. In particular, the comorbidity of SUD and anxiety has been associated with several adverse outcomes, such as increased symptom severity [39] and early relapse to substance use [40]. As suggested by Hser and colleagues [41] the comorbidity of maternal SUD with other mental health disorders may play a critical role in children's developmental outcomes.

It is well-known that the executive functions are involved in the control and regulation of emotional and behavioral processes [12]. Empirical evidence demonstrates that individuals with SUD are more likely to exhibit neuropsychological impairments [20, 42], including impairments in general intelligence and various executive functioning tasks [43], as well as neural abnormalities in frontal lobes, as indicated by imaging studies [44, 45]. Furthermore, research indicates that parental EF could be transmitted through generations [46, 47]. In this regard, Cuevas and colleagues [48] found an association between the EF of mothers and the EF of their 24-month-olds, highlighting the potential effect of maternal caregiving on the development of children's EF.

Only a few studies have investigated the relation between parental neuropsychological functioning and parenting in individuals with SUD [49, 50]. For example, recent research by Håkansson and colleagues [50] investigated the associations between EF and parental reflective functioning in caregivers with SUD, which includes the caregiver's ability to recognize the child's expressions and behaviors as well as the caregiver's appreciation that the inner world of the child may be affected by the inner world and mental state of the parent. The authors found significant associations between these two domains, suggesting that they may both be essential in sensitive caregiving. This may be because adequate functioning of these domains enhances accurate perception, interpretation, and responsiveness to infant cues [49]. To our knowledge, the current study is the first study to examine the associations between parental neuropsychological functioning and the quality of observed parent-child interactions in the context of parental SUD.

We hypothesize that both parental neuropsychological functioning and psychopathology impact the quality of the emotional availability between mother and child. Emotional Availability (EA) focuses on the capacity of a dyad to create a healthy emotional connection and to share a wide range of affective expressions [51, 52]. As such, it provides a useful theoretical frame for investigating parenting and the quality of adult-child relationships [53–55]. EA emphasizes the “emotional features” of parenting, considering a wide range of adult qualities (i.e., sensitivity, structuring, nonintrusiveness, and nonhostility) and taking also into account the child's contribution (i.e., responsiveness and involvement of the parent [51, 52]). In this way, EA allows observers to simultaneously consider different aspects of relationship quality. A variety of prior studies have already focused on EA in the context of parental SUD [21, 26, 56, 57] and in the presence of adult psychopathology [58]. However, to our knowledge, prior studies have not investigated these aspects while also considering the links with parents' neuropsychological functioning. The present study aims to contribute to the extant literature on parents with SUD by examining how parental neuropsychological functioning and psychopathology impact EA during observed mother-child interactions.

## 2. Aims and Hypotheses

The purpose of the present study is to adopt an integrated perspective on parenting in the context of parental Substance Use Disorder (SUD) by investigating the way in which maternal neuropsychological functioning and maternal psychopathology could be associated with observed mother-child EA. First, based on the extant literature, we expect to find lower mean scores in mother-child EA in the context of SUD, as compared to the level of EA observed in normative samples [59]. Second, we hypothesize that, in this group, higher maternal psychopathological symptoms will be associated with lower mother-child EA. Third, we hypothesize that lower maternal neuropsychological functioning will be associated with lower mother-child EA. Finally, we compute a regression model where EA is expected to be predicted by maternal neuropsychological functioning and maternal psychopathology. Our hypothesis is that lower levels in both domains would significantly predict less optimal parenting behaviors.

## 3. Methods

### 3.1. Participants

The study involved 29 women diagnosed with SUD (M age = 30.52 years, SD = 7.37) and their children (16 females and 13 males). The mean age of children was 22.97 months (SD = 28.64). The mothers were attending a rehabilitative program in a Venetian Therapeutic Community. This facility offers residential care to mother-child dyads in the context of maternal SUD or other severe psychiatric illness and provides a comprehensive rehabilitation program over a 2-year period. A mother usually enters the facility after a Juvenile Court decree which implies mandatory intervention for the mother, in order not to lose parental responsibility. The community uses a combined intervention program, integrating both therapeutic (group therapy, individual therapy and mother-child intervention) and educational strategies. For the purposes of the present study, only mothers admitted to the facility due to SUD were included in the present analyses. The diagnosis of SUD was made by expert clinicians on the basis of the patients' anamnesis (patient medical history) and of urine toxicology.

At the time of admission, 64% of the women had interrupted their education before attaining upper secondary qualification. Before entering the facility, most of them lived with their partner (65%) or with their family of origin (25%). Regarding past history, 41.4% reported familiar history for SUD, whereas 51.7% reported significant losses and 60.9% reported past experience of maltreatment (sexual or physical abuse). With respect to personal history of SUD, the subjects reported an early onset in their use of drugs (M = 16 years, SD = 2.74), and most self-reported that onset was due to sensation-seeking (44.8%) or to the attempt to escape personal problems (13.8%). Participants mostly described a pattern of poly-drug use (65.52%), with heroin (65.5%), cocaine (6.9%), and cannabis (6.9%) as primary substances of abuse. Also, 48.3% presented symptoms of drug-related illness, such as Hepatitis C. With respect to pregnancy and motherhood, 50% reported that their pregnancy was desired, and 44.8% reported that they continued to use drugs throughout gestation. As for newborn medical status at delivery, mean values were 39 weeks gestational age (SD = 1.41), 3023.21 grams birthweight (SD = 391.39), 33.19 centimeters for cranial circumference (SD = 1.31), and 48.48 centimeters for length (SD = 1.56). Finally, at delivery, 37.9% of infants presented with Neonatal Abstinence Syndrome (NAS).

### 3.2. Procedures

Data presented in this paper constitutes part of a larger research project approved by the Ethical Committee of the University of Padua. The research protocol was carried out in accordance with the Declaration of Helsinki and with the recommendations of the Code of Ethics approved by the General Assembly of the Italian Association of Psychology. Written informed consent was obtained from mothers prior to the start of any procedures.

Recruitment was initiated after mothers were admitted as patients to the Therapeutic Community. Participation was voluntary and free of charge to the participants. Mothers who agreed to participate underwent an assessment protocol that took place during two one-hour sessions. The assessment included measures aimed at investigating maternal neuropsychological functioning and the presence of psychopathological symptoms. Moreover, mother-child dyads were videotaped during 15 minutes of free-play, and videos were coded using the EA Scales [51].

#### 3.2.1. Maternal Neuropsychological Functioning

Neuropsychological functioning was investigated through the Esame Neuropsicologico Breve-2 [literal translation* Brief Neuropsychological Examination*] (ENB-2; [60]), a comprehensive neuropsychological battery standardized for the Italian population. The battery includes 16 subtests: digit span, immediate and delayed recall prose memory, interference memory at 10 and 30 seconds, trail making test part A and B, token test, word phonemic fluency, abstract reasoning, cognitive estimation, overlapping figures, spontaneous drawing, copy drawing, clock drawing, and ideomotor praxis test. These subtests allow the investigator to assess cognitive domains of attention (trail making test part A and B), memory (digit span, immediate and delayed recall prose memory, and interference memory at 10 and 30 seconds), comprehension (token test), executive functioning (trail making test part B, cognitive estimation, abstract reasoning, phonemic fluency, clock drawing, and overlapping figures), perception (spontaneous drawing, copy drawing), and praxis abilities (ideomotor praxis). The scoring system yields a score for each subtest and to a total score (the Global Cognitive Index (GCI)), describing an individual's overall neuropsychological functioning. The scores can be classified as below average, borderline, and above average, according to established norms. The ENB-2 battery is reported to have good psychometric properties, including adequate test-retest reliability and differential validity in discriminating normative and clinical groups [60, 61]. For the purposes of this study, only the Global Cognitive Index and subtests featuring maternal executive functions (i.e., trail making test-B, cognitive estimation, abstract reasoning, phonemic fluency, clock drawing, and overlapping figures) were considered.

#### 3.2.2. Maternal Psychopathology

The presence of psychopathology in the mother was investigated through the* Symptom Checklist-90 Revised* (SCL-90-R [62]; Italian version by Sarno et al. [63]), a self-report questionnaire designed to evaluate the presence of psychological distress and a range of psychopathological symptoms. The SCL-90-R is composed of 90 items that can be grouped into nine scores along primary symptom dimensions (somatization, obsessive-compulsive, interpersonal sensitivity, depression, anxiety, hostility, phobic anxiety, paranoid ideation, and psychoticism) and three scores that refer to global distress indexes: global psychological distress status (Global Severity Index (GSI)), the total number of symptoms reported (Positive Symptom Total (PST)), and the intensity of perceived distress (Positive Symptom Distress Index (PSDI)). Raw scores can be converted into* T*-scores that can be compared to norms and that aid the identification of severe symptoms. The SCL-90 is well-established as a reliable and valid measure of psychological problems and symptoms, and it is normed on both clinical and nonclinical populations [62]. For the purposes of this study, all symptom scales and distress indexes were considered at a descriptive level, whereas only the GSI was included in the regression model.

#### 3.2.3. Emotional Availability

The quality of videotaped mother-child interactions was assessed using the* Emotional Availability Scales*—4th ed. (*EA Scales*; [51]). This observational coding system is composed of six scales, four that assess the adult (sensitivity, structuring, nonintrusiveness, and nonhostility) and two that assess the child (responsiveness and involvement). The scales consider both behaviors and emotional expressions that occur during the interaction, and they can be used from infancy into adolescence [51, 52].

The* adult sensitivity* scale assesses the adult's capacity to both express healthy and mostly positive range of emotions and to respond to the child quickly and appropriately. Higher scores on this scale indicate that the adult is generally positive in effect, reads the child's cues accurately, responds to cues in a timely manner, is flexible in responsiveness, and behaves in an accepting way toward the child. The* adult structuring *scale evaluates the adult's ability to effectively guide learning and to set age-appropriate limits. Higher scores indicate that the adult offers preventive guidance, is successful in guiding learning, uses both verbal and nonverbal forms of guidance, and enforces appropriate limits. The* adult nonintrusiveness* scale considers the adult's tendency to follow the child's lead, avoid interfering, and permit age-appropriate autonomy. Higher scores indicate that the adult generally follows the child's lead, enters play when welcome, and avoids physical or verbal interferences. The* adult nonhostility* scale assesses the adult's ability to regulate negative emotions and avoid expressing them toward the child or in the presence of the child. This includes both overt hostility, such as ridiculing or threats of separation, and covert hostility, such as impatience, frustration, or irritation. Higher scores indicate that the adult does not express covert or overt hostility, remains cool under stress, does not express frightening or threatening behaviors, and avoids hostile play themes.

The* child responsiveness* scale assesses the child's tendency to express a healthy range of mostly positive emotions and to respond to the adult in a positive and nonanxious manner. Highly responsive children express mainly positive emotions and are comfortable responding to the adult, yet do not compromise their autonomy. Finally, the* child involvement *scale evaluates the child's tendency to invite the adult into interaction and to engage with the adult. Higher scores on this scale indicate that the child frequently reaches out to the adult for emotional and playful exchanges, elaborates upon those exchanges, and rarely uses negative means (e.g., whining) to involve.

Each scale is scored directly on semicontinuous 7 point scales, with higher scores being more optimal. The coding refers to the global quality of the interaction observed rather than on specific behaviors. For each scale, scores between 5.5 and 7 suggest a more healthy interaction. Scores around 4 indicate the presence of inconsistency (i.e., behaviors that are appropriate in some way but that are not fully optimal) or for sensitivity and child responsiveness depending scales also some degree of unhealthy overconnectedness. Scores of 3 or below point out less optimal interactions. Scores lower than 2 indicate low qualities on that dimension.

Moreover, the EA system also provides a measure of attachment security through the Emotional Attachment Zones Evaluation (EA-Z; previously referred to as the Emotional Attachment and Emotional Availability Clinical Screener [51]). The EA-Z categorizes each member of the dyad into one of four attachment “zones,” which conceptually correspond to the four attachment styles [64, 65]. The zones are Emotionally Available, Complicated, Detached, and Problematic (descriptions are patterned after secure attachments, insecure-resistant attachments, insecure-avoidant attachments, and insecure-disorganized attachments, resp.).

### 3.3. Statistical Analyses

Data were analyzed using IBM SPSS statistics, Version 23. Preliminary analyses were run using descriptive statistics (average scores, frequencies, and percentages) to investigate maternal neuropsychological functioning, maternal psychopathology, and EA. Subsequently, Pearson's product-moment correlation coefficient was run to test for associations among neuropsychological functioning, psychopathology, and EA, adopting a multiple testing approach. Given the exploratory purposes of the present study no *p* value adjustment was used, based on the theoretical and methodological assumption that, despite decreasing the risk of Type I errors, such a choice would have increased the risk of making type II errors [66]. Moreover, this choice was supported by the fact that, despite separately, the variables considered have already been tested for associations in previous studies. Finally, this choice was supported empirically, given the medium-to-large effect sizes that were found in the results of the present study [67, 68]. Finally, a 2-step hierarchical multiple regression was conducted in order to predict mother-child EA from maternal neuropsychological functioning and maternal psychopathology.

## 4. Results

### 4.1. Maternal Neuropsychological Functioning

Table 1 reports average scores, standard deviations, and the distribution among normative cut-offs for the ENB-2 scores. With respect to neuropsychological functioning, a significant percentage of mothers presented an impaired cognitive profile, considering both the ENB-2 Global Cognitive Index and maternal executive functions. Specifically, higher rates of impairments were found on the trail making test-B, cognitive estimation, and clock drawing.

### 4.2. Maternal Psychopathology

Table 2 displays average scores, standard deviations, and the distribution among normative cut-offs for the SCL-90-R scores. As is shown in Table 2, participants reported the presence of clinically significant symptoms in different areas, such as paranoid ideation, psychoticism, depression, anxiety, and interpersonal sensitivity. Moreover, this sample had high rates of clinically significant symptoms on the global distress indexes.

### 4.3. Emotional Availability

Table 3 shows average scores, standard deviations, and the distribution of the dyads assessed through the EA Scales and the EA Zones (previously EA2-CS). Mean direct scores for each scale ranged from 3.83 to 4.79, which indicates that, as a group, mothers and children in this sample had relatively low EA. This range of scores was consistent with other studies with drug-exposed samples [28], and it was lower than what is typically found in a normative, low risk sample, where mean scores usually range from 4 to 6 [53, 59]. Please note that this is a descriptive rather than a statistical between this SUD sample and the normative samples reported in other studies. Thus, these results lend support to the first hypothesis, which predicted that these mothers would have lower EA compared to normative samples in the literature.

On the EA-Z, most mothers and children were categorized in the complicated (32.7% and 48.3%, resp.) and detached (14.5% and 37.9%) zones of the scoring system. This indicates that, according to the EA-Z, most dyads presented with an insecure attachment style.

### 4.4. Correlations among Neuropsychological Functioning, Psychopathology, and EA

As shown in Table 4, we found statistically significant correlations between maternal neuropsychological functioning and EA, suggesting that women who performed better on the ENB2 also showed higher EA. Specifically, significant associations were found between maternal executive functioning and global cognitive functioning and EA (maternal sensitivity and nonintrusiveness), indicating that mothers with higher neuropsychological functioning present as more sensitive and less intrusive during mother-child interactions. Furthermore, significant associations were found between maternal neuropsychological functioning and the child EA Scales during mother-child interactions.

Maternal psychopathology was also significantly correlated with EA (Table 5). Mothers who reported higher distress also showed less optimal behaviors during interactions with their children. Again, among the maternal scales, sensitivity was most consistently related to psychopathological symptoms, especially with the GSI. Maternal psychological distress was significantly correlated with the child EA Scales, with children of mothers who reported higher symptoms on the SCL-90-R showing less optimal responsive and involving behaviors. Finally, maternal neuropsychological functioning and maternal psychopathological symptoms were significantly correlated (Table 6). Specifically, higher maternal psychological distress was associated with poorer performance on the ENB2.

### 4.5. The Influence of Neuropsychological Functioning and Psychopathology on EA

Given the strong correlations found among the different measures, we ran a regression model to test the impact of maternal neuropsychological functioning and maternal psychopathology on mother-child EA. A 2-step hierarchical multiple regression (HMR) analysis was conducted including the ENB2 GCI, the SCL-90-R GSI, and EA. Among maternal EA Scales sensitivity was identified as the dependent variable. This choice was due both to the significant bivariate correlations between sensitivity, the ENB2 GCI, and the SCL-90-R GSI and to the fact that, among parental characteristics, sensitivity has been one of the centerpieces of attachment and parent-child interaction research (e.g., [64, 69]). Maternal ENB2 GCl was entered at Step 1 of the regression, and Maternal GSI was entered at Step 2. We included variables of both psychopathology and neuropsychological functioning based on the extant literature highlighting significant associations between these two domains [70].  Table 7 reports regression statistics. The results of the hierarchical multiple regression indicated that, at Step 1, GCI contributed significantly to the prediction of maternal sensitivity *F*(1,25) = 8.62, *p* = 0.007, and accounted for 25.60% of the variance in maternal sensitivity. This indicates that mothers with higher scores on the GCl also showed higher sensitivity during interactions. Introducing the SCL-90 GSI explained an additional 8.7% of the variance in maternal sensitivity. Although the second model resulted in effectively explaining the variance in maternal sensitivity, *F*(2,24) = 6.29, *p* = 0.006, the change in *R*^2^ subsequent to the introduction of the SCL-90 GSI was only marginally significant, Δ*F*(1,24) = 3.20, *p* = 0.086, suggesting that most of the variance in maternal sensitivity was explained by maternal neuropsychological functioning. Thus, mothers who reported higher general distress tended to show lower sensitivity. Together, the two independent variables accounted for the 34.4% of the variance in maternal sensitivity.

## 5. Discussion

The purpose of the present study was to contribute to the extant literature on parenting in the context of SUD, a condition identified as highly risky both for the mother and for the child [19, 21, 22, 26, 27]. Drawing on empirical evidence that highlights the influences of both parental cognitive functioning and psychopathology on observed caregiving behaviors, we proposed an integrated perspective and investigated the way in which maternal neuropsychological functioning and maternal psychopathology were associated with the quality of parent-child relationships [51–53, 55]. More specifically, we investigated these aspects in a group of mothers affected by SUD and their children, hypothesizing to find generally low neuropsychological functioning, high psychopathological symptoms, and low quality mother-child interactions (measured through EA), as well as significant associations between these different domains. As previously highlighted by the literature on parental SUD [71], the participants often reported traumatic histories, characterized by maltreatment, and sexual and/or physical abuse, as well as family histories of SUD. An early onset in the consumption of drugs was described, with frequent patterns of poly-drug use that often continued during pregnancy, leading on some occasions to neonatal adverse consequences at delivery [72].

As hypothesized, significant impairments in maternal neuropsychological functioning were uncovered, with scores that were below the norm on executive functions and overall cognitive profiles, similar to the work of Parolin and colleagues [73] on young adults with SUD. Furthermore, these mothers frequently reported the experience of clinically significant symptoms, in line with previous studies highlighting the presence of higher rates of psychopathology in individuals with SUD [74]. Finally, the dyads in our sample presented with relatively low EA, with average scores around 3 and 4. This indicates the presence of inconsistency and distress during interactions, as well as detachment. Although this result could not be further investigated through statistical analyses, due to the lack of a control group, it is consistent with previous studies [26, 28] that compared mother-child EA in the context of SUD with normative samples. Strikingly, not one of the children in our study was coded as Emotionally Available on the EA-Z, stressing the importance of interventions to help these families [75, 76].

As hypothesized, significant associations were found between maternal EA, maternal neuropsychological functioning, and psychopathology. The strongest associations were found between maternal sensitivity, nonintrusiveness, executive functions, and the Global Cognitive Index, suggesting that mothers who presented with globally higher neuropsychological functioning also demonstrated more sensitive and less intrusive behaviors during mother-child interactions. These links were found especially with ENB2 subtests investigating attention and task switching (trail making test-B), the individual's capacity to answer ambiguous questions drawing from general knowledge of the world (cognitive estimation), and the adequacy of representational, planning, organizational, and coordination abilities (clock drawing). We speculate that these same abilities might be involved in parent-child interactions. To be sensitive to a child's cues, mothers are required to be mentally flexible and to continuously maintain, switch, and update attention on different sources of information. Moreover, mothers are required to mentally represent their child's signals and needs; to disambiguate when needs are not clear; and to plan, organize, and coordinate their responses to the child's cues and behaviors. A deficit in one or more of these abilities could compromise the parent's capacity to be adequately sensitive and responsive to the child's signals. At the same time, similar impairments in these same areas, such as the lack of mental flexibility and difficulties in planning, organizing, and coordinating behavioral responses, as well as the impossibility to update these responses on the basis of the child's feedback (e.g., a child's protest for being interrupted during the ongoing of an activity might lead to parental intrusiveness).

Consistent with our hypotheses and with previous findings [58], EA was also correlated with maternal psychopathology, with those reporting higher distress also showing less optimal parenting behaviors and less optimal interactions with their children. Finally, significant correlations were found between maternal neuropsychological functioning and psychopathological symptoms. These correlational findings were additionally supported by the regression model. Interestingly, most of the variance of maternal sensitivity in our study was explained by maternal neuropsychological functioning and only marginally by maternal psychopathology, suggesting the possibility that, in the case of parental SUD, cognitive impairments might have a higher predictive value on actual caregiving behaviors.

*Limitations*. The first limitation of this study involves the small number of participants, which prevents us from the generalization of the obtained results. Future studies should include a larger number of participants, in order to be able to replicate and further expand the results of the present study. The second limitation involves the lack of a control group. Future research should investigate these questions in normative samples, which are usually characterized by lower rates of cognitive impairments, psychological distress, and higher quality of maternal EA. A third limitation concerns the lack of measures specifically focused on children's executive functioning and psychopathology. The literature has highlighted the fact that experienced parenting behaviors could be associated with the development of children's executive functions (e.g., [46]), suggesting an intergenerational transmission of cognitive processes [13]. Moreover, some studies have highlighted the significant associations existing between the experience of poor-quality parenting and the development of psychopathological symptoms in children. Given that, in our study, we found significant associations between both of these parental measures and children's EA; it would be interesting in the future to explore this aspect, also investigating the impact of parental functioning on children's neuropsychological processes and psychopathology.

*Future Directions*. Future research with larger sample sizes and several time points of measurements could investigate mediational models. For example, we wonder whether there is a pathway from psychological distress to substance abuse (as a self-medication strategy), and then to subsequent neuropsychological impairments (due to persistent drug use) [73, 77]. A different pathway could be from impairments in maternal neuropsychological functioning to impairments in other social/emotional processes that are supported by neurocognitive abilities to some extent (such as empathy), which then may contribute to difficulties in mother-child interactions. For example, a study from Killeen and Teti [78] showed that EA was related to the mother's empathetic response to infant emotional expressions, measured by the activation of her right frontal lobe. Given that the frontal lobes are associated with executive functioning and are impaired in those with SUD [44, 45]. It may be interesting to examine how executive functioning could contribute to lower empathy and difficulties in adequately responding to the child's signals. Studies with larger sample sizes and multiple times points can more closely examine these trajectories in order to extend our understanding of these phenomena as well as help identify areas of prevention and intervention for parents at risk for SUD.

## 6. Conclusions

Parental SUD has been widely linked to dysfunctions in parenting behaviors and parent-child interactions. Furthermore, empirical evidence has highlighted that individuals with SUD often show impairments in neuropsychological functioning and psychological well-being, features which have been previously linked to difficulties in parenting and poor-quality parent-child interactions. To our knowledge, no prior study has examined the links between maternal neuropsychological functioning, psychopathology, and quality of observed EA in this clinical population. To address this gap in the literature, we investigated in a group of mothers with SUD the associations between maternal neuropsychological functioning, maternal psychopathology, and EA, with findings of cognitive/neurological functioning being associated with EA, and to a much lesser extent, with maternal psychopathology being associated with EA. From a clinical point of view, this study leads to important considerations about prevention and clinical interventions for parents with SUD. This clinical group has been previously identified as difficult to treat, especially given the profound impact of drugs on physical, cognitive, and emotional functioning. Herein, we propose an integrated approach that targets different facets of functioning (neuropsychological, mental health, and parent-child relationships). These findings indicate that for parents with SUD, in addition to traditional treatment for addiction and for mental health, it may be critical to target and to improve parent-child EA, which may help to strengthen a mutually rewarding attachment system that challenges the attachment of the mother to drugs, and potentially prevent relapse. Our integrated perspective further considers also the treatment of maternal neuropsychological functioning, with hopes that improvements in cognitive profiles might help to support additional improvements in parenting behaviors. These joint efforts are likely to support not only parental but also child well-being and the well-being of future children for women who have problems with substance use.

## Figures and Tables

**Table 1:** Means, SD, and the distribution among normative cut-offs for the ENB-2.

*Mothers neuropsychological functioning*		
*N* = 29		
	M (SD)	Impairment *N* (%)
*Executive functions*		
Trail making test-B (TMTB)	10.39 (16.12)	11 (37.9)
Cognitive estimation (Cog-Est)	−2.17 (2.96)	18 (62.1)
Abstract reasoning (Ab-Reas)	−.11 (1.47)	7 (24.1)
Phonemic fluency (Ph-Fl)	−.70 (1.06)	10 (34.5)
Clock drawing (Cl-Dr)	−2.78 (4.68)	14 (48.3)
Overlapping figures (Ov-Fig)	−.72 (.99)	6 (20.7)
*Global Cognitive Index (GCI)*	77.79 (9.13)	10 (34.5)

**Table 2:** Means, SD, and distribution of the subjects in the SCL-90-R.

*Mothers' psychopathology* *N* = 27		
*Variable*	M (SD)	Clinical *N* (%)
Somatization (Som)	51.96 (12.10)	8 (29.6)
Obsessive-compulsive (OC)	50.59 (11.26)	7 (25.9)
Interpersonal sensitivity (IS)	53.22 (11.56)	11 (40.7)
Depression (Dep)	55.67 (10.91)	13 (48.1)
Anxiety (Anx)	54.48 (10.92)	11 (40.7)
Hostility (Hos)	53.56 (11.92)	9 (33.3)
Phobic anxiety (Phob)	50.07 (8.26)	6 (22.2)
Paranoid ideation (Par)	55.56 (13.28)	16 (59.3)
Psychoticism (Psy)	59.04 (11.29)	15 (55.6)
*GSI*	55.48 (12.39)	13 (48.1)
*PST*	51.22 (9.15)	11 (40.7)
*PSDI*	59.81 (12.43)	16 (59.3)

GSI = Global Severity Index, PST = Positive Symptom Total, PSDI = Positive Symptom Distress Index.

**Table 3:** Means, SD, and distribution of the dyads on the EA Scales and on the EA-Z.

*Emotional Availability Scales (EA Scales)*			
*N* = 29			
*Mother scales*	M (SD)	*Child scales*	M (SD)
Sensitivity	3.83 (0.74)	Responsiveness	3.50 (0.76)
Structuring	4.03 (0.46)	Involvement	3.36 (0.99)
Nonintrusiveness	4.09 (1.27)		
Nonhostility	4.79 (1.09)		

*Emotional Attachment Zones (EA-Z)*			
*N* = 29			
*Mothers zones*	*N* (%)	*Child zones*	*N* (%)

Emot. Avail.	2 (6.9%)	Emot. Avail.	-
Complicated	18 (32.7%)	Complicated	14 (48.3%)
Detached	8 (14.5%)	Detached	11 (37.9%)
Problematic	1 (1.8%)	Problematic	4 (13.8%)

**Table 4:** Correlations among measures of neuropsychological functioning and maternal behaviors.

	EA Sens	EA Struct	EA Nonint	EA Nonhos	EA Ch. Resp	EA Ch. Invol
*Executive functions*						
TMTB	−.596^**∗∗**^	−.188	−.549^**∗∗**^	−.542^**∗∗**^	−.508^**∗∗**^	−.370^**∗**^
Cog-Est	.104	−.083	−.250	−.153	.034	.019
Ab-Reas	.443^**∗**^	.031	.183	.359	.337	.147
Ph-Fl	.333	−.020	.351	.221	.234	.248
Cl-Dr	.147	−.026	.416^**∗**^	.186	.133	.227
Ov-Fig	.119	−.131	.168	.079	.019	.093
*GCI*	.505^**∗∗**^	.082	.393^**∗**^	.349	.378^**∗**^	.344

TMTB = trail making test-B, Cog-Est = cognitive estimation, Ab-Reas = abstract reasoning, Ph-Fl = phonemic fluency, Cl-Dr = clock drawing, Ov-Fig = overlapping figures, and GCI = Global Cognitive Index. ^*∗*^Correlation is significant at the 0.05 levels (two-tailed). ^*∗∗*^Correlation is significant at the 0.01 levels (two-tailed).

**Table 5:** Correlations among symptoms of psychopathology and maternal behaviors.

	EA Sens	EA Struct	EA Nonint	EA Nonhos	EA Ch. Resp	EA Ch. Invol
Som	−.264	.104	−.353	−.302	−.311	−.288
OC	**−.446** ^**∗**^	−.349	−.275	−.257	**−.493** ^**∗∗**^	** −.483** ^**∗**^
IS	−.329	−.053	−.351	−.136	−.330	** −.381** ^**∗**^
Dep	−.362	−.276	−.288	−.211	** −.397** ^**∗**^	** −.428** ^**∗**^
Anx	−.352	−.045	−.310	−.113	−.264	−.378
Hos	−.232	.220	−.172	−.179	−.123	−.131
Phob	** −.444** ^**∗**^	−.088	−.210	−.275	−.290	−.238
Par	−.326	−.135	−.296	−.294	−.343	−.368
Psy	−.348	−.218	−.221	−.223	−.333	−.335
*GSI*	** −.469** ^**∗**^	−.133	−.377	−.309	** −.426** ^**∗**^	** −.398** ^**∗**^
*PST*	−.367	−.214	** −.400** ^**∗**^	−.139	−.335	** −.431** ^**∗**^
*PSDI*	−.365	.021	−.160	−.313	−.345	−.198

Som = somatization, OC = obsessive-compulsive, IS = interpersonal sensitivity, Dep = depression, Anx = anxiety, Hos = hostility, Phob = phobic anxiety, Par = paranoid ideation, Psy = psychoticism, GSI = Global Severity Index, PST = Positive Symptom Total, and PSDI = Positive Symptom Distress Index. ^*∗*^Correlation is significant at the 0.05 levels (two-tailed). ^*∗∗*^Correlation is significant at the 0.01 levels (two-tailed).

**Table 6:** Correlations among measures of neuropsychological functioning and psychopathology.

	Som	OC	IS	Dep	Anx	Hos	Phob	Par	Psy	GSI	PST	PSDI
*Executive functions*	.145	.065	.175	.186	.117	.060	.176	.109	.206	.210	.183	.176
TMTB	.227	.224	.030	.143	.094	.080	.225	.105	.080	.179	.084	.119
Cog-Est	−.093	−.088	−.022	.003	−.111	−.110	−.136	−.216	−.122	−.126	−.040	−.174
Ab-Reas	−.036	−.183	.074	.017	−.017	−.105	−.244	−.047	−.133	−.098	.044	−.127
Ph-Fl	.106	.145	.073	.098	.216	.030	−.061	−.093	.019	.079	.110	.095
Cl-Dr	−.199	−.141	.038	.028	−.047	−.099	−.064	−.174	−.056	−.112	.007	−.133
Ov-Fig	−.129	−.199	−.207	−.223	−.171	**−.309** ^*^	** −.280** ^**∗**^	−.257	** −.273** ^**∗**^	** −.291** ^**∗**^	−.204	−.213
*GCI*	−.229	−.211	−.084	−.160	−.180	−.185	** −.325** ^**∗**^	−.227	−.250	** −.272** ^**∗**^	−.125	−.243

TMTB = trail making test-B, Cog-Est = cognitive estimation, Ab-Reas = abstract reasoning, Ph-Fl = phonemic fluency, Cl-Dr = clock drawing, Ov-Fig = overlapping figures, GCI = Global Cognitive Index, Som = somatization, OC = obsessive-compulsive, IS = interpersonal sensitivity, Dep = depression, Anx = anxiety, Hos = hostility, Phob = phobic anxiety, Par = paranoid ideation, Psy = psychoticism, GSI = Global Severity Index, PST = Positive Symptom Total, and PSDI = Positive Symptom Distress Index. ^*∗*^Correlation is significant at the 0.05 levels (two-tailed).

**Table 7:** HMR testing relations among maternal cognitive functioning, psychopathology, and EA.

Variable	Maternal sensitivity						
	*B*	SE *B*	*β*	*t*	Δ*R*^2^	Δ*F*	Sig. Δ*F*
Step 1							
ENB2 GCI	.041	.01	.506	2.94	.256	8.62	.007
Step 2							
ENB2 GCI	.031	.01	.382	2.13	.087	3.20	.086
SCL-90 GSI	−.020	.01	−.321	−1.79			

## Data Availability

Data will be available upon request.

## References

[1] Ainsworth M. D. S. (1969). Maternal sensitivity scales. *Journal of Power Sources*.

[2] Biringen Z., Robinson J. (1991). Emotional availability in mother‐child interactions: a reconceptualization for Research. *American Journal of Orthopsychiatry*.

[3] Vizziello G. F., Colucci M. R., Disnan G., Simonelli A. (2003). Psicopatologia dello sviluppo. *Il mulino*.

[4] Simonelli A. (2014). La funzione genitoriale. *Raffaello Cortina Editore*.

[5] Biringen Z. (2000). Emotional availability: Conceptualization and research findings. *American Journal of Orthopsychiatry*.

[6] Belsky J. (1984). The determinants of parenting: a process model.. *Child Development*.

[7] Murray L., Fearon P., Cooper P. (2015). *Identifying Perinatal Depression and Anxiety*.

[8] Nicol-Harper R., Harvey A. G., Stein A. (2007). Interactions between mothers and infants: Impact of maternal anxiety. *Infant Behavior & Development*.

[9] Bridgett D. J., Gartstein M. A., Putnam S. P. (2011). Emerging effortful control in toddlerhood: The role of infant orienting/regulation, maternal effortful control, and maternal time spent in caregiving activities. *Infant Behavior & Development*.

[10] Deater-Deckard K., Sewell M. D., Petrill S. A., Thompson L. A. (2010). Maternal working memory and reactive negativity in parenting. *Psychological Science*.

[11] Pennington B. F., Ozonoff S. (1996). Executive functions and developmental psychopathology. *Journal of Child Psychology and Psychiatry*.

[12] Miller E. K., Cohen J. D. (2001). An integrative theory of prefrontal cortex function. *Annual Review of Neuroscience*.

[13] Bridgett D. J., Burt N. M., Edwards E. S., Deater-Deckard K. (2015). Intergenerational transmission of self-regulation: A multidisciplinary review and integrative conceptual framework. *Psychological Bulletin*.

[14] Crandall A., Deater-Deckard K., Riley A. W. (2015). Maternal emotion and cognitive control capacities and parenting: A conceptual framework. *Developmental Review*.

[15] Chico E., Gonzalez A., Ali N., Steiner M., Fleming A. S. (2014). Executive function and mothering: Challenges faced by teenage mothers. *Developmental Psychobiology*.

[16] Gonzalez A., Jenkins J. M., Steiner M., Fleming A. S. (2012). Maternal early life experiences and parenting: The mediating role of cortisol and executive function. *Journal of the American Academy of Child and Adolescent Psychiatry*.

[17] Dvir Y., Ford J. D., Hill M., Frazier J. A. (2014). Childhood maltreatment, emotional dysregulation, and psychiatric comorbidities. *Harvard Review of Psychiatry*.

[18] Rutherford H. J. V., Booth C. R., Crowley M. J., Mayes L. C. (2016). Investigating the relationship between working memory and emotion regulation in mothers. *Journal of Cognitive Psychology*.

[19] Eiden R. D., Stevens A., Schuetze P., Dombkowski L. E. (2006). Conceptual model for maternal behavior among polydrug cocaine-using mothers: the role of postnatal cocaine use and maternal depression.. *Psychology of addictive behaviors : journal of the Society of Psychologists in Addictive Behaviors*.

[20] Parolin M., Simonelli A. (2016). Attachment theory and maternal drug addiction: The contribution to parenting interventions. *Frontiers in Psychiatry*.

[21] Porreca A., De Palo F., Simonelli A., Capra N. (2016). Attachment representations and early interactions in drug addicted mothers: A case study of four women with distinct adult attachment interview classifications. *Frontiers in Psychology*.

[22] Scaife V. H. (2008). Maternal and paternal drug misuse and outcomes for children: Identifying risk and protective factors. *Children & Society*.

[23] Rutherford H. J. V., Williams S. K., Moy S., Mayes L. C., Johns J. M. (2011). Disruption of maternal parenting circuitry by addictive process: Rewiring of reward and stress systems. *Frontiers in Psychiatry*.

[24] Kim S., Iyengar U., Mayes L. C., Potenza M. N., Rutherford H. J. V., Strathearn L. (2017). Mothers with substance addictions show reduced reward responses when viewing their own infant's face. *Human Brain Mapping*.

[25] Mayes L. C., Feldman R., Granger R. H., Haynes O. M., Bornstein M. H., Schottenfeld R. (1997). The effects of polydrug use with and without cocaine on mother-infant interaction at 3 and 6 months. *Infant Behavior & Development*.

[26] Swanson K., Beckwith L., Howard J. (2000). Intrusive caregiving and quality of attachment in prenatally drug-exposed toddlers and their primary caregivers. *Attachment & Human Development*.

[27] Pajulo M., Savonlahti E., Sourander A., Ahlqvist S., Helenius H., Piha J. (2001). An early report on the mother-baby interactive capacity of substance-abusing mothers. *Journal of Substance Abuse Treatment*.

[28] Salo S., Kivistö K., Korja R. (2009). Emotional availability, parental self-efficacy beliefs, and child development in caregiver-child relationships with buprenorphine-exposed 3-year-olds. *Parenting: Science and Practice*.

[29] Jacobson S. W., Jacobson J. L., Sokol R. J., Martier S. S., Chiodo L. M. (1996). New evidence for neurobehavioral effects of in utero cocaine exposure. *Journal of Pediatrics*.

[30] Tronick E. Z., Weinberg M. K., Seifer R. (2005). Cocaine exposure is associated with subtle compromises of infants' and mothers' social-emotional behavior and dyadic features of their interaction in the face-to-face still-face paradigm. *Developmental Psychology*.

[31] Conway K. P., Compton W., Stinson F. S., Grant B. F. (2006). Lifetime comorbidity of DSM-IV mood and anxiety disorders and specific drug use disorders: Results from the National Epidemiologic Survey on Alcohol and Related Conditions. *Journal of Clinical Psychiatry*.

[32] Lai H. M. X., Cleary M., Sitharthan T., Hunt G. E. (2015). Prevalence of comorbid substance use, anxiety and mood disorders in epidemiological surveys, 1990-2014: A systematic review and meta-analysis. *Drug and Alcohol Dependence*.

[33] Yilmaz O., Dilbaz N. (2016). Complex Comorbidity of Substance Use Disorders with Anxiety Disorders: Diagnosis and Treatment. *New Developments in Anxiety Disorders*.

[34] Swendsen J. D., Merikangas K. R. (2000). The comorbidity of depression and substance use disorders. *Clinical Psychology Review*.

[35] Salloum I. M., Brown E. S. (2017). Management of comorbid bipolar disorder and substance use disorders. *American Journal of Drug and Alcohol Abuse*.

[36] El-Bassel N., Gilbert L., Witte S., Wu E., Chang M. (2011). Intimate partner violence and HIV among drug-involved women: Contexts linking these Two epidemics-challenges and implications for prevention and treatment. *Substance Use & Misuse*.

[37] Dixon L. J., Lee A. A., Gratz K. L., Tull M. T. (2018). Anxiety sensitivity and sleep disturbance: Investigating associations among patients with co-occurring anxiety and substance use disorders. *Journal of Anxiety Disorders*.

[38] Anagnostopoulos A., Ledergerber B., Jaccard R. (2015). Frequency of and risk factors for depression among participants in the swiss HIV Cohort Study (SHCS). *PLoS ONE*.

[39] McEvoy P. M., Shand F. (2008). The effect of comorbid substance use disorders on treatment outcome for anxiety disorders. *Journal of Anxiety Disorders*.

[40] Schellekens A. F. A., de Jong C. A. J., Buitelaar J. K., Verkes R. J. (2015). Co-morbid anxiety disorders predict early relapse after inpatient alcohol treatment. *European Psychiatry*.

[41] Hser Y.-I., Lanza H. I., Li L., Kahn E., Evans E., Schulte M. (2015). Maternal Mental Health and Children’s Internalizing and Externalizing Behaviors: Beyond Maternal Substance Use Disorders. *Journal of Civil Structural Health Monitoring*.

[42] Brand M., Roth-Bauer M., Driessen M., Markowitsch H. J. (2008). Executive functions and risky decision-making in patients with opiate dependence. *Drug and Alcohol Dependence*.

[43] Schmidt T. P., Pennington D. L., Cardoos S. L., Durazzo T. C., Meyerhoff D. J. (2017). Neurocognition and inhibitory control in polysubstance use disorders: Comparison with alcohol use disorders and changes with abstinence. *Journal of Clinical and Experimental Neuropsychology*.

[44] Bechara A., Damasio H., Damasio A. R. (2001). Manipulation of dopamine and serotonin causes different effects on covert and overt decision-making. *Society for Neuroscience - Abstracts*.

[45] Moreno-López L., Catena A., Fernández-Serrano M. J. (2012). Trait impulsivity and prefrontal gray matter reductions in cocaine dependent individuals. *Drug and Alcohol Dependence*.

[46] Bernier A., Carlson S. M., Whipple N. (2010). From external regulation to self-regulation: Early parenting precursors of young children's executive functioning. *Child Development*.

[47] Deater-Deckard K., Sturge-Apple M. L. (2017). Introduction to the special section: Mind and matter: New insights on the role of parental cognitive and neurobiological functioning in process models of parenting. *Journal of Family Psychology*.

[48] Cuevas K., Deater-Deckard K., Kim-Spoon J., Wang Z., Morasch K. C., Bell M. A. (2014). A longitudinal intergenerational analysis of executive functions during early childhood. *British Journal of Developmental Psychology*.

[49] Håkansson U., Halsa A., Söderström K., Skårderud F., Øie M. G. (2015). Keeping mind in mind: Mentalizing and executive functioning in substance-abusing infant mothers: Effect on dyadic relationship and infant outcome. *Substance Abuse: Research and Treatment*.

[50] Håkansson U., Söderström K., Watten R., Skårderud F., Øie M. G. (2017). Parental reflective functioning and executive functioning in mothers with substance use disorder. *Attachment & Human Development*.

[51] Biringen Z. (2008). *The Emotional Availability (EA) scales*.

[52] Biringen Z., Robinson J., Emde R. (1998). *The Emotional Availability (EA) Scales*.

[53] Biringen Z., Derscheid D., Vliegen N., Closson L., Easterbrooks M. A. (2014). Emotional availability (EA): Theoretical background, empirical research using the EA Scales, and clinical applications. *Developmental Review*.

[54] Biringen Z., Easterbrooks M. A. (2012). Emotional availability: Concept, research, and window on developmental psychopathology. *Development and Psychopathology*.

[55] Saunders H., Kraus A., Barone L., Biringen Z. (2015). Emotional availability: Theory, research, and intervention. *Frontiers in Psychology*.

[56] Flykt M., Punamäki R.-L., Belt R. (2012). Maternal representations and emotional availability among drug-abusing and nonusing mothers and their infants. *Infant Mental Health Journal*.

[57] Salo S., Politi J., Tupola S. (2010). Early development of opioid-exposed infants born to mothers in buprenorphine-replacement therapy. *Journal of Reproductive and Infant Psychology*.

[58] Trapolini T., Ungerer J., McMahon C. (2008). Maternal depression: Relations with maternal caregiving representations and emotional availability during the preschool years. *Attachment & Human Development*.

[59] Licata M., Kristen S., Sodian B. (2016). Mother-Child Interaction as a Cradle of Theory of Mind: The Role of Maternal Emotional Availability. *Social Development*.

[60] Mondini S., Mapelli D., Vestri A., Bisiacchi P. S. (2003). *Esame Neuropsicologico Breve*.

[61] Mondini S., Mapelli D., Vestri A., Arcara G., Bisiacchi P. S. (2011). *L’Esame Neuropsicologico Breve-2*.

[62] Derogatis L. (1975). *SCL-90-R: Symtom Checklist-90-R: Administration, scoring, and procedures manual*.

[63] Sarno I., Preti E., Prunas A., Madeddu F. (2011). *SCL-90_R Symtom Checklist-90-R Adattamento Italiano*.

[64] Ainsworth M. D. S., Blehar M. C., Waters E., Wall S. N. (1978). *Patterns of attachment: A psychological study of the strange situation*.

[65] Main M., Solomon J. (1990). Procedures for identifying infants as disorganized/disoriented during the Ainsowrth Strange Situation. Attach. Presch. years Theory. *Res. Interv*.

[66] Feise R. J. (2002). Do multiple outcome measures require p-value adjustment?. *BMC Medical Research Methodology*.

[67] Cohen J. (1992). Statistical power analysis. *Current Directions in Psychological Science*.

[68] Hojat M., Xu G. (2004). A Visitor's Guide to Effect Sizes – Statistical Significance Versus Practical (Clinical) Importance of Research Findings. *Advances in Health Sciences Education*.

[69] Biringen Z., Damon J., Grigg W. (2005). Emotional availability: Differential predictions to infant attachment and kindergarten adjustment based on observation time and context. *Infant Mental Health Journal*.

[70] Snyder H. R., Miyake A., Hankin B. L. (2015). Advancing understanding of executive function impairments and psychopathology: bridging the gap between clinical and cognitive approaches. *Frontiers in Psychology*.

[71] Stocco S., Simonelli A., Capra N., De Palo F. (2012). Research and intervention for drug-addicted mothers and their children: New Perspectives. *Addictions - From Pathophysiology to Treatment*.

[72] Patrick S. W., Schumacher R. E., Horbar J. D. (2016). Improving care for neonatal abstinence syndrome. *Pediatrics*.

[73] Parolin M., Simonelli A., Mapelli D., Sacco M., Cristofalo P. (2016). Parental substance abuse as an early traumatic event. Preliminary findings on neuropsychological and personality functioning in young drug addicts exposed to drugs early. *Frontiers in Psychology*.

[74] De Palo F., Capra N., Simonelli A., Salcuni S., Di Riso D. (2014). Parenting quality in drug-addicted mothers in a therapeutic mother—child community: the contribution of attachment and personality assessment. *Frontiers in Psychology*.

[75] Berlin L. J., Shanahan M., Appleyard Carmody K. (2014). Promoting supportive parenting in new mothers with substance-use problems: A pilot randomized trial of residential treatment plus an attachment-based parenting program. *Infant Mental Health Journal*.

[76] Suchman N. E., DeCoste C., Castiglioni N., McMahon T. J., Rounsaville B., Mayes L. (2010). The mothers and toddlers program, an attachment-based parenting intervention for substance using women: Post-treatment results from a randomized clinical pilot. *Attachment & Human Development*.

[77] Parolin M., Simonelli A., Cristofalo P. (2017). Neuropsychological deficits in young drug addicts. *Heroin Addiction and Related Clinical Problems*.

[78] Killeen L. A., Teti D. M. (2012). Mothers' frontal EEG asymmetry in response to infant emotion states and mother-infant emotional availability, emotional experience, and internalizing symptoms. *Development and Psychopathology*.

